# Factors Correlated with the Quality of Life after Total Knee Arthroplasties: A Literature Review

**DOI:** 10.5704/MOJ.2403.001

**Published:** 2024-03

**Authors:** A Sunadi, Nursalam, Mustikasari, I Krisnana, ND Kurniawati

**Affiliations:** 1 Department of Nursing, Universitas Respati Indonesia, Jakarta, Indonesia; 2 Department of Nursing, Universitas Indonesia, Depok, Indonesia; 3 Department of Nursing, Universitas Airlangga, Surabaya, Indonesia

**Keywords:** total knee arthroplasty, factor, quality of life, post-operative, outcome

## Abstract

**Introduction:**

Total Knee Arthroplasty (TKA) has been widely reported to improve outcomes and quality of life (QoL) in patients with knee osteoarthritis (KOA), but there are still 15 - 20% of patients still experience pain, physical limitations, and other complications after TKA. Therefore, it is necessary to identify various factors that correlate with QoL from current evidence. The objective is to review the literature on factors that correlate with QoL in patients who underwent TKA.

**Materials and methods:**

A literature search was conducted on five databases, i.e. ProQuest, CINAHL, Medline, Embase, and Scopus, using the following keywords: total knee arthroplasty (TKA), post-operative, quality of life (QoL), and outcome. There were no restrictions on the research design.

**Results:**

This review found 14 articles (7 prospective studies and 7 retrospective studies) involving 15,972 patients who underwent TKA, with an age range of 32 - 94 years. All articles reported improvement in QoL after TKA. The review revealed 30 factors, of which 15 factors were significantly correlated with QoL after TKA. The factors were grouped into four types: demographic, socioeconomic, clinical, and psychosocial factors.

**Conclusion:**

Information regarding factors that correlate with QoL after TKA can be used for directing treatment and discharge planning according to the patient's factors.

## Introduction

Knee osteoarthritis (KOA) is a common musculoskeletal disorder that is characterised by chronic pain and functional disability^1-3^. The global prevalence of KOA is as high as 16%, with an incidence of up to 203 per 10,000 person-years^4^. KOA is the main cause of pain, decreased self-care ability, and physical limitations in the elderly, which strongly reduces quality of life (QoL) and has an impact on the patient's psychosocial condition. The prevalence of KOA in patients aged <45 years also continues to increase^5-7^. Patients with KOA often experience decreased self-care abilities and disability due to joint pain, stiffness, and limited activities, which have an impact on decreasing QoL and causing various socioeconomic problems^6,8,9^.

Along with the increasing prevalence and adverse effects of KOA, the demand for Total Knee Arthroplasties (TKA) is also increasing^5,10^. TKA is the procedure of choice in advanced KOA that does not respond to non-surgical therapies and is the most effective surgical procedure for reducing pain and correcting deformity and is expected to improve QoL in patients with KOA^8,11^.

TKA was first performed 40 years ago^12^, and several previous studies have shown that 80-85% of patients with KOA have good outcomes after TKA^13-15^. However, 15-20% of patients still complain of persistent pain, poor joint function, post-operative infections, and other complications^8,11,16^. Thus, it is necessary to identify various factors that contribute to improving or worsening QoL based on current evidence. Several previous studies have identified the association between various factors and QoL after TKA but have shown inconsistent results. This literature review summarises and examines various factors, both correlated and not significantly correlated with QoL after TKA, based on previous studies.

## Materials and Methods

Search on five databases i.e. ProQuest, CINAHL, MEDLINE, Embase, and Scopus. The article search uses Boolean operators, such as 'AND' and 'OR' by combining several key words, namely total knee arthroplasty (TKA), factor, post-operative, quality of life, outcome. Article searches were limited to full-text articles published in the last 10 years and in English. There were no restrictions on the research design.

Quality of life after TKA was the main outcome studied. The extracted data included the year, country, study design, sample size, age, sex ratio, instruments used, follow-up, and factors identified as correlated with outcomes after TKA. Selected articles were presented in tabular form and the data were analysed narratively. All search results were initially reviewed by the first three authors (A, N, and M) based on their titles and abstracts.

## Results

In our review, we found 14 articles consisting of 7 prospective studies and 7 retrospective studies involving 15,972 patients who underwent TKA (Table I). The ages of the range from 32 - 94 to years. Thirteen of the 14 articles (except Snell *et al*^17^) showed a higher proportion of female to men, ranging from 61% to 85%.

**Table I:** Study Characteristics

Author, Years	Country	Design	Sample Size	Age (y)	M:F	Outcome measure	Follow up (d, m, y)	Factors identified*
González-Sáenz- de-Tejada *et al*^2^, 2023	Spain	Prospective cohort study	471	67.9±8.6	24,6:75,4	WOMAC, SF-36	6m, 10y	Age, gender, BMI, having social support, civil status, comorbidity, days in hospital, OA severity, post-operative complication, readmission (6 months), complication from hospital discharge, rehabilitation from discharge, baseline QoL
Chang *et al*^8^, 2022	China	Prospective multicentre observational study	404	40	91:313	KOOS-PS	7d, 1m, 3m, 6m	Sex, age, marital status, education, smoker, alcohol consumption, income, medical insurance, duration of illness, expected duration of recovery, trauma, hospital stay, daily exercise, BMI
Papakostidou *et al*^11^, 2012	Yunani	Prospective study	204	69.17±6.69	42:162	WOMAC, KSS, CES-D10, VAS for pain	3m, 6m, 1y	Preintervention pain, preintervention function, age, BMI, place of residence, level of education, social support
Snell *et al*^17^, 2020	Switzerland	Retrospective cross-sectional study	409	68.3±8.7	54%:46%	WHOQOL: EUROHIS-QOL 8-item index, OKS	6m	Age, gender, procedure type, ASA, comorbidities, BMI
Mawarikado *et al*^18^, 2022	Japan	Retrospective study	156	74.1±7.3	35/121	KOOS, mGES	3m, 1y	Age, sex, BMI, FTA, implant type, rest pain, walking pain, ROM of extension, ROM of flexion, IKES, walking speed, mGES
Siviero *et al*^19^, 2020	Italy	Prospective study	132	67.9±8.6	35:97	SF-12, WOMAC	3m	Age, sex, education, BMI, depressive symptoms, cognitive impairment, comorbidity, current smoker, current alcohol user
Bindawa^23^, 2018	Saudi Arabia	Longitudinal study (progression and incident cohort)	4674	63.0±8.8	46%:54%	KOOS-QoL	4y	Age, gender, race, education, marital status, income per year, health care coverage, Charlson comorbidity index (CCI), depressive symptoms, BMI
Al-Omran^21^, 2014	Saudi Arabia	Retrospective cross-sectional study	52	64.75±7.90	9:43	The study does not use scoring system. The measurement of QoL is based on the pain and walking pre- operatively and post-operatively	-	Age, gender, pre-operative night pain, pain on walking, post-operative night walking, post-operative pain on walking
Clement *et al* 22, 2022	UK	Retrospective study	3127	71.5 (32-94)	456:2671	EQ-5D	2y	Patella resurfacing, gender, mean age, BMI, ASA grade, Oxford score
Kanopka *et al*^23^, 2018	New York	Retrospective study	5398	66.9±9.5	38%:61,2%	EQ-5D, SF-6D	2y	Age, sex, BMI, CCI, ASA score, pre-operative EQ-5D score
Charles-Lozoya *et al*^24^, 2019	Mexico	Prospective cross-sectional study	378	71 (51-89)	38.3%:61.7%	SF-36, CENASEM	5y	Obesity, depression
Jiménez Ortiz *et al*^25^, 2019	Spain	Prospective study	260	70.8	67:193	KSS, VAS, HADS, WOMAC	1y	Pre-operative anxiety, pre-operative depression
Utrillas-Compaired *et al*^26^, 2014	Spain	Prospective cohort study	202	3±6.3	30,7%:69,3%	WOMAC, KSS, VAS, HAD	1y	Pre-operative psychologic distress
Schwartsmann *et al*^27^, 2017	Brazil	Retrospective cohort study	60	60-80	15:45	KSS	1.5m	Patellar resurfacing

*) Factors in bold have a significant correlation with the outcome Abbreviations - ASA: American Society of Anaesthesiologists, BMI: Body Mass Index, CCI: Charlson Comorbidity Index, CENASEM: Mexican Health and Age Study, CES-D10: Centre for Epidemiological Studies Depression Scale, EQ-5D: European Quality of Life Five Dimension, EUROHIS: European Health Interview Survey, FTA: Femorotibial angle, HADS: Hospital Anxiety and Depression Scale, IKES: Isometric Knee Extension Strength, IKS: Imperial Knee Score, KOOS-PS: Knee injury and Osteoarthritis Outcome Score-Physical Function Shortform, KSRS: Knee Society Rating System, KSS: Knee Society Score, mGES: modified Gait Efficacy Scale, OKS: Oxford Knee Score, QoL: Quality of Life, SF-12:12-Item Short Form Health Survey, SF-36:36-Item Short Form Health Survey, TKA: Total Knee Arthroplasty, WHOQOL: The World Health Organization Quality of Life, WOMAC: Western Ontario and McMaster’s Universities, VAS: Visual Analogue Scale

QoL and outcome measurements were performed using various types of instruments. Some studies have used generic QoL instruments that include general physical, mental, and psychosocial aspects. Meanwhile, other instruments focus on physical and functional aspects. There were 15 types of instruments used to assess outcomes after TKA: Western Ontario and McMaster's Universities (WOMAC), 36-Item Short Form Health Survey (SF-36), 12-Item Short Form Health Survey (SF-12), European Health Interview Survey-Quality of Life (EUROHIS-QOL), European Quality of Life Five Dimension (EQ-5D), Knee Society Score (KSS), Charlson Comorbidity Index (CCI), Mexican Health and Age Study (CENASEM), Centre for Epidemiological Studies Depression Scale (CES-D10), Hospital Anxiety and Depression Scale (HAD), Imperial Knee Score (IKS), Knee Injury and Osteoarthritis Outcome Score-Physical Function Shortform (KOOS-PS), Oxford Hip and Knee Score (OHKS), visual analog scale (VAS), and modified Gait Efficacy Scale (mGES). Of these instruments, WOMAC, KSS, and SF-12/SF-36 are the most frequently used.

The results of the study identified 30 factors that were correlated with QoL and outcomes after TKA. These factors were grouped into four types: demographic, socioeconomic, clinical, and psychosocial. Table II shows 15 of the 30 factors found to be significantly correlated with QoL, that is, age, sex, race, education, income, current alcohol use, Body Mass Index (BMI)/obesity, comorbidities, American Society of Anaesthesiologists (ASA) grade, daily exercise, preoperative QoL, pain and pre-operative function, readmission, depressive symptoms, and self-efficacy.

**Table II:** Factors Correlated to Quality of Life after TKA

Factor type	Significant factors	Non-significant factors
Demographic factors	1. Age^2,18,20,21,22^ 2. Sex/gender^18,19,22,23^ 3. Race^20^	1. Marital status/civil status^2,8,20^ 2. Place of residence^11^ 3. Medical insurance/health care coverage^8,20^
Socioeconomic factors	4. Education^19^ 5. Income^8,20^	4. Current smoker^8,19^ 5. OA severity^2^
Clinical factors	6. Current alcohol user^19^ 7. BMI/obesity^2,17,19,20,22,23^ 8. Comorbidity^2,17,19,20,23^ 9. ASA grade^22,23^ 10. Daily exercise^8^ 11. Pre-operative QoL^2,23^ 12. Pre-operative pain and function^11,18,22^ 13. Readmission^2^	6. Duration of illness^8^ 7. Expected duration of recovery^8^ 8. Trauma^8^ 9. Patella resurfacing^22,27^ 10. Pain on walking^21^ 11. Post-operative night walking^21^ 12. Post-operative pain on walking^21^ 13. Days in hospital^2,8^
Psychosocial factors	14. Depressive symptoms/depression^19,20,24-26^ 15. Self-efficacy^18^	14. Cognitive impairment^19^ 15. Social support^2,11^

Abbreviations - ASA: American Society of Anaesthesiologists, BMI: Body Mass Index, OA: osteoarthritis

## Discussion

In our review, all studies demonstrated that patients with KOA experienced improved QoL and outcomes after TKA. Papakostidou *et al*^11^ showed that 95% of patients with KOA experienced a significant improvement in QoL after TKA from the first three months, whereas 5% of patients experienced some mild symptoms 12 months after surgery.

The follow-up period varied in each study, but the positive impact of TKA on QoL appeared after three months and continued significantly for up to five years when compared to conditions before surgery. Mawarikado *et al*^18^ revealed that significant and stable improvements occurred 12 weeks post-TKA, especially in terms of physical function and pain. This is similar to the study by Siviero *et al*^19^, who reported that the QoL of patients improved three months after surgery, confirming that TKA can provide a substantial improvement in the QoL of patients with end-stage KOA. In particular, the specific symptoms of KOA (pain, stiffness, decreased function) are better than those before TKA^28^.

Papakostidou *et al*^11^ found that three weeks after surgery, despite improvements in pain and depression levels, function was still unsatisfactory. In another study, Bruyere *et al*^29^ has observed that from six weeks after surgery to the end of follow-up (seven years), there was continuous improvement in the physical and emotional dimensions of both the SF-36 and WOMAC. The study by Shah *et al*^30^ also showed that there was a significant difference in the SF-36 score before and six months after TKA, especially in the physical health and mental health components.

We found a correlation between age and QoL in 9 of 15 studies, with inconsistent results, where 5 of 9 studies showed a significant correlation. The studies by Bindawas^20^ and Al-Omran^21^ showed that age was significantly correlated with QoL in TKA patients post-operatively. A study by Goh *et al*^31^ showed that patients younger than 50 years who underwent TKA experienced significant improvement in their QoL and were satisfied with their surgery. According to González-Sáenz-de-Tejada *et al*^2^, this could be due to the fact that elderly people is often associated with more comorbid conditions, greater weakness, loss of physiological function and, in some cases, development of sarcopenia, a discrepancy that may result in a poorer outcome.

These results are different from those of the systematic review by Santaguida *et al*^32^, who concluded that age is not a prognostic factor for outcomes after TKA. Elderly people are more sedentary, have low expectations of TKA, and accept their condition as a consequence of the aging process. Murphy *et al*^33^ revealed that the inconsistency of the correlation between age and QOL arises from a lack of consistency in defining young and old age and using different methodologies to evaluate QoL, resulting in failure to accurately estimate the impact of age on QoL.

We also found a correlation between gender and QoL in 8 of 15 studies, with inconsistent results, where 4 of 8 studies showed a significant correlation. Women with osteoarthritis may experience more pain and are more sensitive to pain, resulting in a lower QoL^34^. Cherian *et al*^35^ reported that both men and women experienced an increase in QoL after TKA; however, men were better than women, especially in the function and activity domains. In addition, Neuburger *et al*^36^ revealed that women can also be exposed to socioeconomic disadvantages compared to men, which has an impact on the recovery process and QoL after TKA.

We found only one study that identified a relationship between race and QoL after TKA^20^. Ibrahim^37^ has proven that there is a racial disparity in the decision to perform TKA. Most patient preferences are shaped by the outcome of joint surgery expectations, which vary by race. In a study by Groeneveld *et al*^38^, black patients had lower expectations of TKA and were more likely to expect a longer hospital stay, experience more pain, and find it more difficult to ambulate post-TKA.

Among the four studies that identified a correlation between education and QoL after TKA, one study showed a significant correlation^19^. Zhou *et al*^39^ showed that the higher the level of education, the better the patient outcome. KSS scores increased as the level of education increased. Patients with higher education levels had a better understanding of rehabilitation goals, and their knowledge was significantly higher than that of patients with lower education levels. Furthermore, in a study by Kong *et al*^40^, educational level was an independent risk factor for post-operative pain in TKA patients. Pua *et al*^41^ stated that pain relief was better in TKA patients with a higher educational level than in those with a lower educational level. Kong *et al*^40^ also stated that it was natural that the level of education was an independent factor affecting the self-efficacy of patients who underwent rehabilitation after TKA.

We found two studies that showed a significant correlation between income and QoL after TKA^8,20^. Singh *et al*^42^ found that patients with lower incomes had better pain outcomes than those with higher incomes at two years post-TKA. There was more improvement in knee function and a trend toward less activity restriction after primary TKA in low-income patients than in those with higher incomes. These findings confirm that lower income is not a risk factor for poor post-TKA outcomes. Future studies should investigate the reasons for the better outcomes after TKA in low-income patients.

We found only one study that showed a significant correlation between current alcohol use and QoL after TKA^19^. Siviero *et al*^19^ showed that high alcohol intake leads to more postoperative complications, including perioperative joint injury (PJI). Furthermore, a study by Lavernia *et al*^43^ showed that patients who did not drink alcohol experienced a higher change in SF-36 scores than patients who drank alcohol. After surgery, patients who did not drink alcohol experienced greater improvements in their health status. Alcohol consumption mediates inflammation or host tissue response, which can hinder functional recovery and overall health status.

We found six studies that showed a significant correlation between BMI and obesity and QoL after TKA^2,17,19,20,22,23^. Patients with grade II obesity have significantly reduced QoL after TKA. Patients with a BMI <35 have the opportunity to experience better post-operative QoL^22^. Xu *et al*^44^ found that both obese and non-obese patients had significant improvements in function and QoL after surgery; however, obese patients tended to experience smaller improvements in QoL 10 years after TKA.

We found a correlation between comorbidities and QoL in five of the 14 studies with consistent results^2,17,19,20,23^. Comorbidity significantly reduced QoL after TKA. Comorbidities increase the risk of hospital readmission in the short term and affect function, pain, and QoL in the long term^2,19,20^. In the study by Snell *et al*^17^, the most common comorbidities were musculoskeletal conditions, such as arthritis in other joints and other musculoskeletal pain, cardiovascular conditions, such as hypertension, and coronary heart disease. Additionally, Tew *et al*^45^ showed that one-fifth of the patients who underwent TKA developed diabetes. Patients with diabetes, especially women, showed a significantly lower QoL than those without diabetes.

We found two studies that showed a significant correlation between ASA grade and QoL after TKA^2,17^. Clement *et al*^22^ argued that the higher the ASA grade, the lower the QoL, because patients are more likely to suffer complications as a result of comorbidities. Kanopka *et al*^23^ also revealed that ASA grade is a very important factor for predicting the quality-adjusted life year (QALY), which is a generic measure of health-related quality of life (HRQoL). Furthermore, Schaeffer *et al*^46^ reported that patients with ASA score ≥73 have 2.9 times chance of experiencing readmission, then it is also reported that patients with ASA score ≥3 experience an increased risk of post-operative complications, thus ASA score is a potential target in planning interventions to improve QoL and lower costs in arthroplasty patients.

We found only one study that identified a correlation between daily exercise and QoL after TKA, namely the study by Chang *et al*^8^, which showed that patients who exercised <30 minutes and ≥30 minutes daily experienced better outcomes compared to those who did not exercise. Regular exercise protects and improves knee function in KOA patients and accelerates post-operative recovery.

We found two studies that showed a significant correlation between pre-operative QoL and QoL after TKA^2,23^. Mawarikado *et al*^18^ reported that pre-operative QoL had an impact on QoL at 3 and 12 months after TKA. Papakostidou *et al*^11^ also reported that QoL before surgery, especially in the pain and function domains, was a strong determinant of QoL 12 months after surgery.

Patients with worse pre-operative HRQoL may not achieve the same final HRQoL as the other patients. This suggests that if HRQoL is reduced pre-operatively, patients may not enjoy the same level of post-operative HRQoL as they might have hoped for prior to the decline, that is, they may experience a greater increase but not achieve the same final HRQoL as they had hoped^22,47^. In the long-term follow-up conducted by González-Sáenz-de-Tejada *et al*^2^ showed that the higher the pre-operative WOMAC score, the greater the improvement experienced by patients at 10 years follow-up, and from 6 months to 6 years, the patient's score remained stable, except for function, and the scores decreased even though the decrease was only slight.

We found at least three studies that showed a significant correlation between pre-operative pain and function, and QoL after TKA^11,18,22^. TKA is the most effective surgical treatment option for relieving pain and improving function in patients who are unresponsive to conservative therapy^48^. A study by Linberg *et al*^49^ showed that in patients who experienced moderate pre-operative pain, their pain decreased for nine months after TKA, and in patients who experienced high preoperative pain, their pain increased in the first three months and then decreased slightly for nine months after TKA. Furthermore, a study by Desmeules *et al*^50^ showed that patients who experienced higher pre-operative pain were significantly associated with poor HRQoL six months after undergoing TKA.

We found only one study that identified a correlation between readmission and QoL after TKA. Patients who were readmitted within six months had a lower post-TKA HRQoL^2^. A study by Dahlgren *et al*^51^ showed that 26.2% of patients experienced readmission after TKA and 51% of these patients continued to experience complaints that required additional hospitalisation. A study by Phruetthiphat *et al*^52^ showed that BMI, metabolic equivalents (METSs), and CCI were significantly associated with 30-day readmission in post-TKA patients.

We found at least five studies that showed a significant correlation between depressive symptoms/depression and QoL after TKA^19,20,24-26^. Charles-Lazoya *et al*^24^ found that patients with depression had low scores across all SF-36 domains; therefore, the detection of depression before surgery should be an integral part of the treatment protocol in patients with knee arthritis to increase the benefit of TKA. Jimenez Ortiz *et al*^25^ also reported that pre-operative depression appeared to be correlated with poor outcomes in functional knee scores. While it is difficult to determine whether the severity of osteoarthritis leads to increased rates of depression or vice versa, depression affects the perception of symptoms, which will result in patients feeling the effects of their disease and ultimately having poorer scores.

We found only one study that identified a correlation between self-efficacy and QoL after TKA^18^. Self-efficacy is defined as the level of confidence in how well a person can perform an action to produce a result. In a study by Mawarikado *et al*^18^, self-efficacy was measured using the mGES, which is an instrument used to assess patient perceptions regarding their level of confidence in walking. The mGES score has been proven to affect all KOOS subscales at 3 and 12 months after TKA.

Furthermore, Mawarikado *et al*^18^ revealed that self-efficacy is a predictor of physical activity. Confidence in walking pre-operatively affects post-operative walking and other activities. Many patients undergo TKA because knee pain reduces their walking ability. Even under such circumstances, patients with high pre-operative walking confidence were more likely to be motivated to perform post-operative physical therapy exercises and ADL than those with low self-confidence. Consequently, it is credible that a higher preoperative mGES score contributes to an increase in HRQOL 12 months post-TKA.

The results of this review should be interpreted in light of several limitations. In this review, we did not assess the quality of the methodology or risk of bias. In addition, due to the heterogeneity in research methods, instruments used, domains measured, and follow-up period, it is difficult to determine the magnitude of the contribution of each factor to the QoL of patients who underwent TKA.

Restrictions on searching articles only in the last 10 years, in English and full-text, allow for literature that is relevant to the topic of the review but is not included in this review. In addition, the results of our review show that there are inconsistencies in the factors that are significantly correlated with QoL (such as age, gender, education, and BMI). The authors determined that a factor had a significant correlation if there was at least one study that showed a significant correlation of that factor with QoL or outcome after TKA.

## Conclusion

TKA can improve the QoL of patients with advanced KOA, but there is still a possibility that TKA will not have a positive impact as expected by patients because patients have factors that contribute to worsening their QoL. Information regarding factors, especially those that correlate significantly with QoL after TKA, can be considered for directing treatment according to the patient factors. In addition, post-treatment discharge planning can be adjusted according to patient factors, so that it is expected to have a long-term positive impact on reducing the potential complications of TKA and improving the QoL of patients after TKA. Improved understanding of QoL and its correlated factors can provide valuable information for the effectiveness of interventions and lead to better patient care.
